# Cetuximab in combination with irinotecan/5-fluorouracil/folinic acid (FOLFIRI) in the initial treatment of metastatic colorectal cancer: a multicentre two-part phase I/II study

**DOI:** 10.1186/1471-2407-9-112

**Published:** 2009-04-14

**Authors:** Jean-Luc Raoul, Jean-Luc Van Laethem, Marc Peeters, Catherine Brezault, Fares Husseini, Laurent Cals, Johannes Nippgen, Anja-Helena Loos, Philippe Rougier

**Affiliations:** 1 Digestive Oncology Department, Centre E Marquis, 35062 Rennes cedex, France; 2 Gastroenterology Department, Hôpital Universitaire Erasme, Route de Lennik 808, B-1070 Brussels, Belgium; 3 Ghent University Hospital, De Pintelaan 185, 9000 Ghent, Belgium; 4 Hôpital Cochin, Paris, France; 5 Hôpital Pasteur, 39 Ave de la Liberté, 68024 Colmar cedex, France; 6 Oncology Service, Hôpital Font Pré, BP1412, 83056 Toulon cedex, France; 7 Merck KGaA, Darmstadt, Germany; 8 Hôpital Ambroise Paré, 9 Avenue Charles de Gaulle, 92100 Boulogne cedex, France

## Abstract

**Background:**

This study was designed to investigate the efficacy and safety of the epidermal growth factor receptor (EGFR) inhibitor cetuximab combined with irinotecan, folinic acid (FA) and two different doses of infusional 5-fluorouracil (5-FU) in the first-line treatment of EGFR-detectable metastatic colorectal cancer.

**Methods:**

The 5-FU dose was selected on the basis of dose-limiting toxicities (DLTs) during part I of the study. Patients received cetuximab (400 mg/m^2 ^initial dose and 250 mg/m^2^/week thereafter) and every 2 weeks irinotecan (180 mg/m^2^), FA (400 mg/m^2^) and 5-FU (either low dose [LD], 300 mg/m^2 ^bolus plus 2,000 mg/m^2 ^46-hour infusion, n = 7; or, high-dose [HD], 400 mg/m^2 ^bolus plus 2,400 mg/m^2^; n = 45).

**Results:**

Only two DLTs occurred in the HD group, and HD 5-FU was selected for use in part II. Apart from rash, commonly observed grade 3/4 adverse events such as leucopenia, diarrhoea, vomiting and asthenia occurred within the expected range for FOLFIRI. Among 52 patients, the overall response rate was 48%. Median progression-free survival (PFS) was 8.6 months (counting all reported progressions) and the median overall survival was 22.4 months. Treatment facilitated the resection of initially unresectable metastases in fourteen patients (27%): of these, 10 patients (71%) had no residual tumour after surgery, and these resections hindered the estimation of PFS.

**Conclusion:**

The combination of cetuximab and FOLFIRI was active and well tolerated in this setting. Initially unresectable metastases became resectable in one-quarter of patients, with a high number of complete resections, and these promising results formed the basis for the investigation of FOLFIRI with and without cetuximab in the phase III CRYSTAL trial.

## Background

Worldwide, colorectal cancer (CRC) is the third most commonly diagnosed malignancy.[1] In Europe alone in 2006, there were an estimated 412,900 new cases and over 207,000 deaths from the disease.[2] The addition of irinotecan[3,4] or oxaliplatin[5,6] to 5-FU/FA-based regimens, has improved the efficacy of treatment. Combinations of irinotecan and 5-FU/FA have increased overall survival (OS) times to approximately 20 months.[3,4,7,8] These improvements in efficacy, however, have come at a price of increased toxicity. Diarrhoea and neutropenia are commonly observed with irinotecan-based regimens, although they are generally manageable.[3]

A positive correlation between tumour response rate and metastatic resection rate, has been demonstrated in metastatic CRC (mCRC) patients receiving neoadjuvant chemotherapy.[9] Resection remains the best chance of cure, with five-year survival rates of up to 60% having been reported in carefully selected patients.[10] With the advent of more effective chemotherapy and a corresponding improvement in response rates, the incidence of complete resection is increasing. New therapeutic options, which will increase both the magnitude of response and the number of responders, to render incurable disease curable, are being sought. [11-15]

Cetuximab, an IgG1 monoclonal antibody, specifically targets the epidermal growth factor receptor (EGFR) with high affinity. The EGFR is widely expressed by a range of tumours, including CRC [16-18] where it has been reported to be associated with a slightly poorer prognosis.[19,20] Cetuximab blockade of EGFR results in inhibition of tumour growth, invasion, metastasis and angiogenesis. [21-23] Cetuximab has demonstrated efficacy benefits in patients with mCRC that have progressed on irinotecan-containing therapy, either in combination with irinotecan[16,24] or as a single agent.[18,25]

This two-part, phase I/II study was designed to evaluate the safety and efficacy of cetuximab in combination with FOLFIRI in CRC patients with previously untreated unresectable metastatic disease, and to define a dosing regimen for further investigation exploring two different doses of 5-FU.

## Methods

### Eligibility criteria

Patients ≥18 years of age were eligible for study entry if they had: histologically confirmed stage IV colorectal adenocarcinoma with unresectable metastases, immunohistochemically detectable EGFR expression in the primary tumour or metastases; ≥ one uni-dimensionally measurable lesion outside irradiated areas, a life expectancy of ≥3 months; a Karnofsky Performance Status (KPS) of ≥60; completion of previous adjuvant chemotherapy >4 weeks prior to study entry and recovery from the effects of previous chemotherapy/radiotherapy; adequate haematological, renal and hepatic function. The main exclusion criteria were: radiotherapy or surgery <4 weeks prior to study entry; any previous chemotherapy for mCRC; and evidence of brain metastases.

The protocol, and any protocol amendments, for this multicentre study were approved by independent ethics committees for each centre. The study was conducted in accordance with the Declaration of Helsinki (1996). All patients provided written informed consent.

### Study design

**Figure 1:**
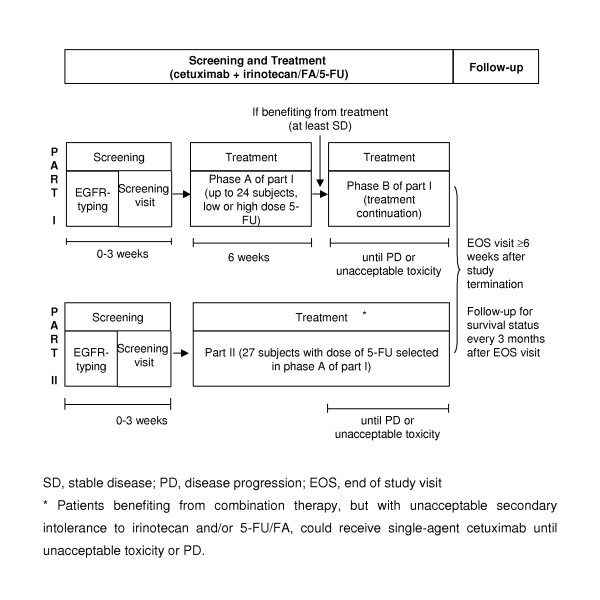
**Study design**.

This was a multicentre, uncontrolled, open-label, two-part, phase I/II study, which enrolled patients between October 2001 and June 2003. All patients received 2-weekly cycles of a combination of weekly cetuximab and 2-weekly chemotherapy, comprising irinotecan and either high-dose (HD, 2,800 mg/m^2^) or low-dose (LD, 2,300 mg/m^2^) 5-FU and FA. In the initial part of the study, cohorts of patients were sequentially assigned to treatment with either LD or HD 5-FU. Patients benefiting from treatment (at least stable disease [SD]) after 3 cycles (6 weeks, phase A) continued with the treatment at the same dose of 5-FU until disease progression (PD) or unacceptable toxicity (phase B, Figure 1). The choice of 5-FU dose for additional patients in part II of the study was based on the evaluation of dose-limiting toxicities (DLTs) observed in phase A of part I. Patients showing a benefit (at least stable disease [SD]) after 3 cycles of treatment, continued combination therapy until PD or unacceptable toxicity. Patients benefiting from combination therapy, but with unacceptable secondary intolerance to irinotecan and/or 5-FU/FA, could receive single-agent cetuximab until unacceptable toxicity or PD.

### Objectives

The primary objective of the study was to define the highest safe dose of intermittent infusional 5-FU out of the two given doses in combination with cetuximab and irinotecan for the first-line therapy of mCRC, based on DLTs and adverse events. The secondary objectives included the evaluation of: best overall response, which was defined as the best confirmed response (Response Evaluation Criteria in Solid Tumors [RECIST] criteria: complete response, CR, or partial response, PR, persisting ≥28 days) from the start of treatment until PD; duration of response (the time from the first assessment of a confirmed CR or PR to the first documentation of PD, or death on study, death within 60 days after the last tumour assessment or the first dose of cetuximab, whichever occurred first); all responses were reviewed by one investigator (P. Rougier). Progression-free survival time (PFS) was also evaluated (the time from the first dose of cetuximab until the first observation of PD or death due to any cause within 60 days after the last tumour assessment). In this PFS estimation, the patients who benefited from secondary surgery for metastases were censored at the date of the surgery, although none had PD at this date. Censoring of patients due to study discontinuation for reasons other than PD (due to planned resection of residual disease or other reasons) was termed informative censoring. We subsequently defined a progression/recurrence-free survival (PRFS) time, which used the date of recurrence after surgery as the date of progression for the resected patients. The OS time was also a secondary objective (the time from the day of the first dose of cetuximab to death).

### Treatment

Cetuximab was administered intravenously (iv) as an initial 2-hour infusion of 400 mg/m^2 ^(day 1), including a test dose of 20 mg, followed by weekly 1-hour infusions of 250 mg/m^2^. Irinotecan, 5-FU and FA were administered 2-weekly. Irinotecan (180 mg/m^2 ^iv) was administered at least 1 hour after the end of the cetuximab infusion. FA (400 mg/m^2 ^iv) was administered as a 2-hour infusion on day 1, prior to 5-FU infusion. 5-FU was administered following irinotecan and FA at one of two doses: LD, 300 mg/m^2 ^bolus plus 2,000 mg/m^2 ^as a continuous 46-hour infusion; HD, 400 mg/m^2 ^bolus plus 2,400 mg/m^2 ^as a continuous 46-hour infusion.

### Assignment to dosing levels – part I

**Figure 2:**
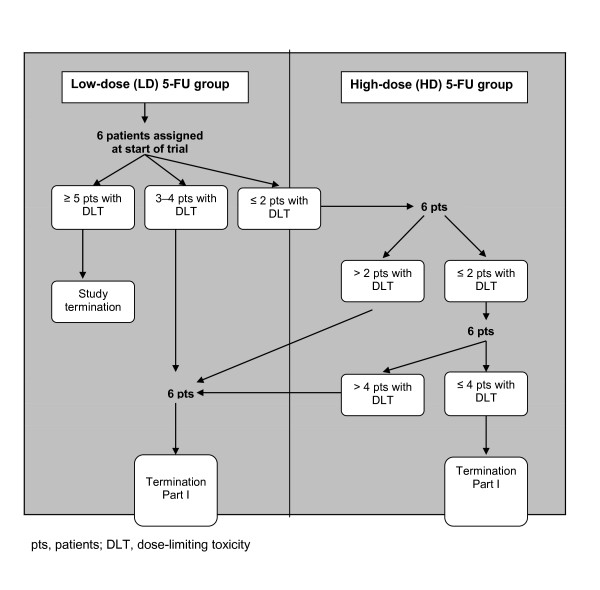
**Dose assignment scheme to LD and HD 5-FU in part I**.

Two dose levels for 5-FU were considered: LD 5-FU and HD 5-FU. Cohorts of six patients were enrolled starting with LD 5-FU. A cohort with HD 5-FU was to be started if fewer than three DLTs were observed during the first 6 weeks (phase A-DLT evaluation period) (see Figure 2 for details of the 5-FU escalation procedure). If three or four DLTs were observed in the LD cohort, another cohort of six patients was enrolled into the LD group and if more than four DLTs occurred, then the trial had to be stopped (Figure 2). If more than two DLTs occurred in the first HD cohort, another cohort of LD 5-FU was started. If not more than two patients experienced a DLT in the first HD cohort, another HD cohort was started. If more than four patients experienced DLTs under HD after two cohorts of HD, patients were enrolled in another LD cohort. During phase A, additional patients had to be recruited to replace patients who discontinued for reasons other than toxicity prior to completion of phase A.

### Dosing modifications

In the case of grade 3 skin reactions, cetuximab could be interrupted for up to two consecutive infusions. After an infusion delay of 1 week and following resolution of symptoms to ≤grade 2, cetuximab could be resumed at the same dose in patients experiencing their first occurrence of grade 3 skin toxicity or at a reduced dose of 200 or 150 mg/m^2 ^(for second and third occurrences, respectively). Treatment in these patients was discontinued in the event of a fourth occurrence of a grade 3 reaction. In patients with grade 3 skin reactions that failed to resolve to ≤grade 2 following an initial infusion delay of 1 week, patients could delay treatment for a further week but were discontinued if more than two consecutive infusions were delayed, or if there was any further occurrence of grade 3 skin toxicity during treatment. Cetuximab-associated infusion-related reactions were managed as follows: grade 1/2, increase of infusion time up to 4 hours and, if this had no effect, consider patient withdrawal from the study; grade 3/4, immediate cessation of cetuximab infusion and patient withdrawal from study.

Treatment was delayed by 1 to 2 weeks to allow for recovery from chemotherapy-associated toxicities. Dose reductions of irinotecan and 5-FU were made for toxicities including: neutropenia (>grade 3), febrile neutropenia, thrombocytopenia or other organ toxicity (>grade 2), mucositis, diarrhoea, or constipation (>grade 1). Dose reductions of 5-FU were made for hand-foot syndrome (HFS) >grade 2.

### Dose-limiting toxicities

The DLT evaluation period was limited to the first 6 weeks of treatment in part I, phase A of the study. A DLT was defined as the occurrence of at least one of the following toxicities according to National Cancer Institute-Common Toxicity Criteria (NCI-CTC) version 2.0: grade 4 neutropenia, leucopenia or thrombocytopenia; > grade 3 skin reactions; > grade 2 febrile neutropenia, infection with neutropenia, anaemia, diarrhoea, mucositis, hepatic toxicity, creatinine levels, or any other medically relevant treatment-related organ toxicity; any drug-related adverse event/s which required treatment interruption.

### Pre-treatment assessments and response and toxicity evaluations

EGFR expression was determined by an independent pathologist using the Dako EGFR pharmDx™ (Dako, Glostrup, Denmark) on tumour tissue available prior to the study. Tumours were considered EGFR-detectable if any tumour cell demonstrated any expression of the receptor. Independently of DLTs, adverse events, graded according to NCI-CTC version 2.0, were recorded throughout the study. Tumour response was based on investigator tumour measurements (reviewed by the principal investigator) of target and non-target lesions, using computed tomography (CT) or magnetic resonance imaging (MRI). The same imaging technique was to be used in the same patient throughout the study. Imaging was performed at baseline, at weeks 6 and 12 and then every 3 months.

### Statistical analyses

Three populations are described: the DLT population (ie all patients from part I who completed the first 3 cycles [phase A] or who stopped treatment because of a DLT); the intention-to-treat (ITT) population (all patients included in the study); and the safety population (all patients who received at least one dose of cetuximab). Assessment of the primary study objective was performed on the DLT population. Adverse events recorded in part I, phase A of the study were also included in the overall adverse event assessments. Efficacy results were based on investigators' assessments of patients in the ITT population. Continuous variables were summarised using descriptive statistics (N, mean, median, standard deviation, minimum, maximum, and first and third quartile). Qualitative variables were summarised by counts and percentages. Kaplan-Meier methods were applied to all time-to event variables. All data were pooled across centres and overall estimates of the treatment effects are provided. No formal statistical tests were performed.

As a substantial number of patients were censored for PFS (56%), sensitivity analysis of the PFS time of these patients was performed, and we have estimated the PRFS time, which included follow-up data. As documentation of a PD date was available in the follow-up period for most of the patients, all follow-up data underwent a thorough review of additional information, such as second-line treatment and treatment after surgery to explore evidence of PD before the reporting date. A PD was considered as an event for this analysis if the date was given before the start of a new line of anti-cancer treatment. Chemotherapy administered following surgery was not considered as a new treatment line.

## Results

### Patient characteristics

A total of 52 patients were enrolled in the study (23 in part I and 29 in part II) and comprise the ITT and safety populations: seven received LD 5-FU and 45 received HD 5-FU. There were 23 patients in the DLT population in part I, phase A (n = 7, LD 5-FU; n = 16, HD 5-FU). There were three major protocol violations in part I, as a result of which two patients were replaced for DLT evaluation. However, one patient enrolled into the HD 5-FU group received LD 5-FU for 7 cycles and subsequently received the correct HD 5-FU and was therefore included as an addition to the initially planned number of six patients in this group. Baseline demographic and pre-treatment characteristics were similar in the two dose groups (Table 1).

**Table 1:** Patient and disease characteristics and prior treatments

Characteristics	LD 5-FU (n = 7)	HD 5-FU (n = 45)	Total (n = 52)
Gender, M/F (%)	57/43	64/36	63.5/36.5

Median age, years [range]	66 [49–73]	61 [25–77]	61 [25–77]

Median KPS [range]	100 [80–100]	100 [60–100]	100 [60–100]

Median duration of disease, months [range]	1.4 [1–25]	1.5 [0–84]	1.5 [0–84]

Primary tumour site (%):			
Colon	86	67	69
Rectum	14	33	31

Previous adjuvant chemotherapy (%)	14	20	19
Number of involved organs (%) ≤ 2	100	93	94
Normal WBC at baseline (%)	100	96	96
Normal LDH at baseline (%)	57	49	50
Normal ALP at baseline (%)	29	36	35
EGFR status (%):^a^			
0–≤10	43	42	42
>10–≤20	14	13	14
>20–≤35	29	13	15
>35	14	31	29

^a^Percentage of positive cells; LD = low-dose; HD = high-dose; KPS = Karnofsky Performance Status; WBC = white blood cell; LDH = lactate dehydrogenase; ALP = alkaline phosphatase; EGFR = epidermal growth factor receptor.

All patients discontinued study treatment. Three patients received cetuximab after the end of study discontinuation of chemotherapy. The most common reason for discontinuation of treatment was PD (35%). However, a high proportion of patients (27%) discontinued study treatment without PD due to the fact that treatment had rendered their initially unresectable disease operable, allowing surgery of the residual lesions. Three patients (6%) discontinued due to adverse events (one with LD 5-FU and two with HD 5-FU).

### Treatment

Assessment of the relative dose intensity (RDI) showed that RDIs of ≤80% were recorded for most patients: cetuximab (90%), irinotecan (81%) and 5-FU (92%). A total of 83% of patients received further lines of antineoplastic treatments after the study.

### DLTs and recommended dose level

In part I, phase A of the study, no DLTs were reported in the LD group. Two patients in the HD group experienced DLTs (13%) (Table 2). The safety profile of HD 5-FU was considered to be acceptable based on these results and thus was pursued as the recommended dose in part II of the study.

**Table 2:** Dose-limiting toxicities in part I, phase A (DLT population)

Events	No. of patients (%)	
	**LD 5-FU (n = 7)**	**HD 5-FU (n = 16)**

No. patients with any event	0 (0)	2 (13)

Leucopenia or neutropenia grade 4	0	1 (6)

Occurrence of any drug-related AE that required treatment interruption within the first six weeks	0	1 (6)*

LD = low-dose; HD = high-dose; AE = adverse event. Seven patients were recruited in the low-dose 5-FU arm due to an overlap in the enrolment of two patients.*Anaphylactic reaction.

### Tolerability

Treatment was generally well tolerated and adverse events were those expected with the treatments and the underlying disease. The most frequently reported adverse events (any grade) were diarrhoea (77%), asthenia (75%), nausea (67%) and rash (52%). Grade 3/4 adverse events were reported in 65% of patients and the most frequent were leucopenia, acne-like rash, diarrhoea, vomiting and asthenia (Table 3). Cetuximab-related grade 3/4 adverse events were reported in 48% of patients.

**Table 3:** Grade 3/4 adverse events occurring in at least two patients in the safety population

Preferred term	Number of patients				Number (%) patients	
	**LD 5-FU** **(n = 7)**		**HD 5-FU** **(n = 45)**		**Total** **(n = 52)**	
	
	**Grade 3**	**Grade 4**	**Grade 3**	**Grade 4**	**Grade 3**	**Grade 4**

Leucopenia	2 [2]	0	8 [8]	[1]	10 (19)	1 (2)

Diarrhoea	0	0	6 [5]	0	6 (12)	0 (0)

Vomiting	1 [1]	0	5 [3]	0	6 (12)	0 (0)

Rash	2 [2]	0	4 [4]	0	6 (12)	0 (0)

Acne	2 [2]	0	3 [3]	0	5 (10)	0 (0)

Asthenia	2 [2]	0	3 [1]	1	5 (10)	1 (2)

Intestinal obstruction	0	0	4	1	4 (8)	1 (2)

Abdominal pain	0	0	3	0	3 (6)	0 (0)

Mucous membrane disorder	0	0	3 [3]	0	3 (6)	0 (0)

Dyspnoea	0	0	3 [3]	1	3 (6)	1 (2)

Atrial fibrillation	0	0	2	0	2 (4)	0 (0)

Deep thrombophlebitis	0	0	2 [1]	0	2 (4)	0 (0)

Gamma glutamyl transpeptidase increased	0	0	2 [1]	0	2 (4)	0 (0)

Hypokalaemia	0	0	2	0	2 (4)	0 (0)

Liver function test abnormal	0	0	2 [1]	0	2 (4)	0 (0)

Skin disorder	0	0	2 [2]	0	2 (4)	0 (0)

Thrombosis	0	0	2	0	2 (4)	0 (0)

Urinary tract infection	0	0	2	0	2 (4)	0 (0)

Weight loss	0	0	2 [1]	0	2 (4)	0 (0)

Square brackets indicate the number of events related to study treatment. LD = low-dose; HD = high-dose.

Serious adverse events (SAEs) were reported in 50% of patients. Cetuximab or chemotherapy was discontinued due to adverse events in three patients (6%). Four (8%) patients died within the period from randomisation to 30 days after the last dose of cetuximab, two (4%) of PD, one of disease-related liver complications, and the other of pneumonia as an unrelated illness. There were no deaths considered to be treatment-related.

### Efficacy

In the overall study population, a best confirmed overall response rate of 48% was reported, with rates of 57% and 47% in the LD and HD groups, respectively (Table 4). Five (10%) additional patients had unconfirmed responses, two (4%) of which were not confirmed because surgery of the lesions was performed. There were no complete responses. The disease control rates were 87%, 86% and 87%, overall and in the LD and HD groups, respectively. The median duration of response was 9.9 months in the overall study population, 7.7 months in the LD population and 9.9 months in the HD population.

**Table 4:** Response (ITT population)

	LD 5-FU (n = 7)	HD 5-FU (n = 45)	Total (n = 52)
Response rate (%)			

Best overall response rate [95% CI]^a^	57 [18, 90]	47 [32, 62]	48 [34, 62]

Stable disease	29	40	39

Progressive disease	14	9	10

Not assessable	0	4	4

Disease control rate (CR + PR + SD) (%) [95% CI]	86 [42, 100]	87 [73, 95]	87 [74, 94]

Median duration of response, days [95% CI]	7.7 [2.9, 12.2]	9.9 [8.1, 13.6]	9.9 [8.1, 11.4]

^a^All partial responses. LD = low-dose 5-FU; HD = high-dose 5-FU; CI = confidence intervals; CR = complete response; PR = partial response; SD = stable disease

**Figure 3:**
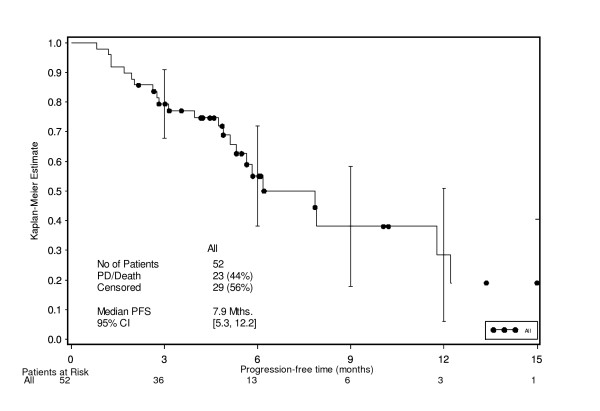
**Progression-free survival time – ITT population**.

**Figure 4:**
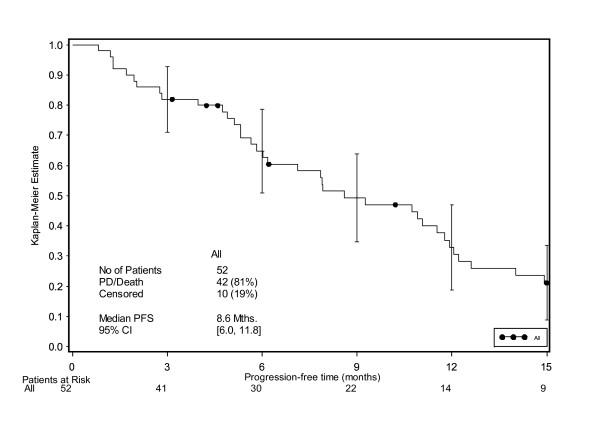
**Progression-free survival time – including follow-up information on PD**.

The median PFS was estimated as 7.9 months overall [95% CI 5.3, 12.2] (Figure 3), and 5.8 [95% CI 2.8, NE] and 7.9 months [95% CI 5.1, 11.8] within the LD and HD populations, respectively. However a substantial proportion of patients in this analysis (56%) did not develop measurable PD but were subject to informative censoring due to study discontinuation (27% due to planned resection of residual disease, 15% due to patient request for a treatment pause following good response to treatment). Therefore, a further exploratory analysis of PRFS, which included follow-up data and recurrence after resection, was performed. The proportion of censoring in this analysis decreased to 19% and the remaining censored observations were considered unlikely to be subject to informative censoring. With the inclusion of the PRFS into the analysis, the median PFS was found to be 8.6 months (Figure 4).

**Figure 5:**
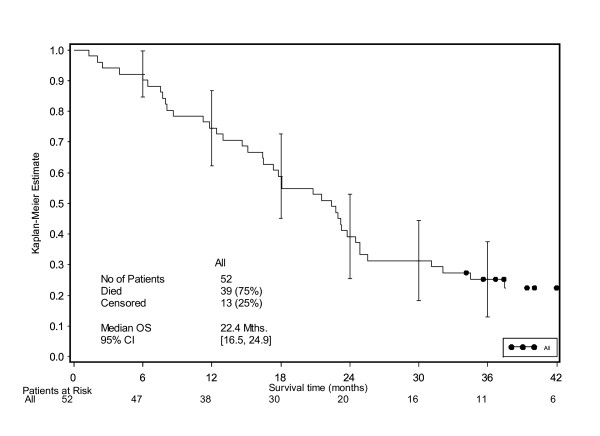
**Overall survival time**.

The median OS was 22.4 months [95% CI 16.5, 24.9] over the whole population (Figure 5), and 18.1 months [95% CI 8.7, 24.9] and 22.6 months [95% CI 16.5, 25.6] in the LD and HD populations, respectively.

At least nine (17%) patients were still alive after a follow up period of more than 4 years.

### Complete resection of initially unresectable disease

Over one-quarter (n = 14, 27%) of patients underwent surgery of residual disease with curative intent: 21% for liver metastases, 2% for lung and 4% for metastases at other sites. There was no residual tumour after surgery in 71% of these patients. Of the remaining patients, one had microscopic and two, macroscopic residual lesions, and the final patient was not assessable.

## Discussion

The initial part of this two-part study confirmed that weekly cetuximab could be safely combined with 2-weekly irinotecan and the usual dose of 5-FU in the modified FOLFIRI-regimen[3,7] (400 mg/m^2 ^bolus plus 2,400 mg/m^2^)/FA, which formed the high dose 5-FU-regimen of this study. The second part of the study demonstrated that this combination of cetuximab and the FOLFIRI regimen was associated with promising efficacy and good tolerability in the first-line treatment of mCRC. Of the 45 patients treated with the HD 5-FU regimen, 47% achieved an objective response. The median PFS for all 52 patients, according to an exploratory analysis adding in PD information from the follow-up, was 8.6 months (7.9 months according to the planned analysis with only on-study events). The survival time of 22.4 months reported in this study is favourable compared with those reported for different schedules of FOLFIRI alone in the first-line treatment of patients with mCRC (17.4 months and 20.1 months), many of whom received similar second-line therapy (FOLFOX and FOLFIRI) to the patients in this study.[3,7] The survival time also compares well with the 20.3 months reported by adding the vascular endothelial growth factor (VEGF) inhibitor, bevacizumab, to first-line bolus irinotecan/5-FU/FA.[26]

In the present study, treatment was generally well tolerated. The majority of patients were able to receive more than 80% of the planned dose of each drug and there were no treatment-related deaths. The addition of cetuximab to the 2-weekly regimen of FOLFIRI induced few additional side effects and did not aggravate the typical grade 3/4 toxicities associated with this chemotherapeutic regimen.

The data from this study, suggesting the benefits of adding cetuximab to combinations of irinotecan and 5-FU in the first-line treatment of mCRC, are supported by results from two other phase I/II studies. In an initial study, the combination of cetuximab with irinotecan (125 mg/m^2^/week) and bolus 5-FU (500 mg/m^2^/week)/leucovorin led to an overall response rate of 48% [95% CI 29, 68].[27] The most frequently reported grade 3/4 adverse events were diarrhoea and neutropenia (28% for each). More recently, Folprecht et al[17] investigated the tolerability and safety of cetuximab with a combination of irinotecan and the AIO weekly infusional 5-FU/FA regimen in a phase I/II trial in 21 patients with previously untreated, EGFR-detectable mCRC. 5-FU was administered weekly as a 24-hour infusion at low (1,500 mg/m^2^, n = 6) or high (2,000 mg/m^2^, n = 15) doses, with FA at 500 mg/m^2 ^and irinotecan at 80 mg/m^2^. The lower 5-FU dose of 1,500 mg/m^2 ^was recommended for future investigation. A confirmed response was reported for 67% [95% CI 47, 87] of patients and the median survival time was 33 [95% CI 20, not reached (NR)] months. Combinations of cetuximab and 5-FU/FA/oxaliplatin have also shown efficacy in the first-line treatment of mCRC. [28-30] In a study of 43 patients, cetuximab plus FOLFOX-4 led to a response rate of 72% [95% CI 56, 85]. The median overall survival time was 30 [95% CI 18, 34] months.[28]. In another study, among 49 patients receiving cetuximab and FUFOX (weekly oxaliplatin plus 5-FU/FA), there was a confirmed response rate of 57% [95% CI 42, 71] and a median overall survival time of 28.2 [95% CI 15, NR] months.[30]

Resection of colorectal metastases is recognised as improving the long-term survival of patients with metastatic disease, and is the only chance of cure for some patients. Response rate is a good indicator of resectability.[9] In this study, 27% of patients had initially unresectable metastatic disease rendered resectable by treatment: nearly three-quarters of these patients had complete resections. The results seen in this study support those reported with a combination of cetuximab and either FOLFOX (23%)[28] or the AIO schedule of 5-FU/FA and irinotecan (24%).[17] These secondary resections have to be considered a good indicator of treatment efficacy. However, estimation of PFS may be difficult with current study designs where patients discontinued from study treatment for curative surgery have no assessment of PD and, thus, are censored at the time of the last on-study tumour assessment. This was a major reason for conducting sensitivity analyses on PRFS, including reliable follow-up information. However, even with these modifications, PFS may be biased because of patients stopping treatment for a pause because of a good response (15% in this study). These two points underline the need to develop more sensitive endpoints better able to demonstrate the true benefit of antineoplastic treatments, particularly as targeted therapies may moderately increase response rates but also improve the quality of the response and reduce the risk of progression. While the concept of PRFS must be validated by other studies, it may help in part to answer these questions.

Since the completion of this phase I/II trial, the use of molecular biomarkers to predict response to treatment has become the focus of much interest. Mutations in the KRAS gene have been identified in a randomised trial in mCRC as being predictive of resistance to the IgG2 EGFR-targeted monoclonal antibody panitumumab [31]. Data from single-arm studies suggest that KRAS mutations are also predictive for resistance to cetuximab, both in the first-line setting and in patients who have failed previous chemotherapy. [32-35] This has been recently confirmed in randomised trials in which cetuximab has been administered first-line to mCRC patients in combination with FOLFIRI [36,37] or FOLFOX [38] versus chemotherapy alone or to chemotherapy-refractory patients in combination with best supportive care versus best supportive care alone.[39] This is an important step towards the more effective tailoring of treatment with cetuximab.

## Conclusion

In summary, the results from the present phase I/II study suggest that the combination of cetuximab and FOLFIRI is an active and well tolerated treatment option for previously untreated mCRC that allows secondary resections without increased morbidity. On the basis of these phase I/II results, the phase III randomised CRYSTAL trial, comparing the efficacy of FOLFIRI (at the same doses used in this study) with and without cetuximab, was initiated in EGFR-detectable mCRC. First efficacy data from the CRYSTAL trial show good activity and consistency with this study, with a 47% response rate and a median PFS of 8.9 months in the cetuximab plus FOLFIRI arm: both response rate and PFS were significantly improved with the addition of cetuximab to FOLFIRI compared with FOLFIRI alone.[37]

## Competing interests

1. Authors' declaration of personal interests:

(i) P Rougier has participated in advisory boards or at symposia as a speaker or expert for Amgen, Merck KGaA, Pfizer, Roche and sanofi-aventis. M Peeters has served as a speaker, a consultant or an advisory board member for Merck KGaA, sanofi-aventis and Roche.

(ii) J Nippgen and A-H Loos are employees of Merck KGaA.

## Authors' contributions

PR conceived and designed the study, entered patients into the trial, analysed the data and drafted the manuscript. JLR, JLV, MP, CB, FH, LC entered patients into the trial and reviewed the manuscript. JN, AHL analysed the data and reviewed the manuscript. All authors read and approved the final manuscript.

## Pre-publication history

The pre-publication history for this paper can be accessed here:

http://www.biomedcentral.com/1471-2407/9/112/prepub
